# Influence of Demographic and Lifestyle Variables on Plasma Magnesium Concentrations and Their Associations with Cardiovascular Risk Factors in a Mediterranean Population

**DOI:** 10.3390/nu12041018

**Published:** 2020-04-08

**Authors:** Rocío Barragán, Juan Llopis, Olga Portolés, Jose V. Sorlí, Oscar Coltell, Lorenzo Rivas-García, Eva M. Asensio, Carolina Ortega-Azorín, Dolores Corella, Cristina Sánchez-González

**Affiliations:** 1 Department of Preventive Medicine and Public Health, School of Medicine, University of Valencia, 46010 Valencia, Spain; rocio.barragan@uv.es (R.B.); olga.portoles@uv.es (O.P.); jose.sorli@uv.es (J.V.S.); eva.m.asensio@uv.es (E.M.A.); Carolina.Ortega@uv.es (C.O.-A.); 2 CIBER Fisiopatología de la Obesidad y Nutrición, Instituto de Salud Carlos III, 28029 Madrid, Spain; oscar.coltell@uji.es; 3 Department of Physiology, School of Pharmacy, University of Granada, 18071 Granada, Spain; jllopis@ugr.es (J.L.); lorenrivas_lrg@hotmail.com (L.R.-G.); crissg@ugr.es (C.S.-G.); 4 Department of Computer Languages and Systems, Universitat Jaume I, 12071 Castellón, Spain

**Keywords:** magnesium, diabetes, cholesterol, mediterranean diet, mediterranean population

## Abstract

Several studies have shown that a low magnesium (Mg) intake in the diet is associated with greater cardiovascular risk and greater risk of diabetes. However, the results are not consistent in all populations. To minimize the biases derived from diet measurement, more objective biomarkers of magnesium status have been proposed. Although there is still no ideal biomarker for Mg, several studies have shown that plasma Mg concentrations could be a relatively acceptable biomarker for cardiovascular risk assessment. However, further studies are required to better characterize this marker in different populations. Our aim was to analyze the association between plasma Mg concentrations (measured through inductively coupled plasma mass spectrometry (ICP-MS)) methods, and cardiovascular risk factors in individuals from a general Mediterranean population (aged 18–80 years). The influence of demographic and lifestyle variables, including adherence to the Mediterranean diet, on plasma Mg concentrations was analyzed. The mean Mg level of the population studied was 0.77 ± 0.08 mmol/L, the prevalence of hypomagnesemia (<0.70 mmol/L) being 18.6%. We did not find any statistically significant differences between plasma Mg concentrations and sex, age, tobacco smoking and total adherence to the Mediterranean diet (*p* > 0.05). We found a statistically significant association between plasma Mg concentrations and the prevalence of type-2 diabetes (0.77 ± 0.08 mmol/L in non-diabetics versus 0.73 ± 0.13 mmol/L in diabetics; *p* = 0.009). Despite the low prevalence of type-2 diabetes in this population (11.24% in subjects with hypomagnesemia versus 3.91%, in normomagnesemia; *p* = 0.005), hypomagnesemia was associated with greater odds of being diabetic in comparison with normomagnesemia (OR = 3.36; *p* = 0.016, even after adjustment for sex, age, obesity, and medications). On the other hand, no statistically significant association of plasma Mg concentrations with obesity, hypertension, fasting triglycerides, HDL-cholesterol or uric acid was found. However, in contrast to what was initially expected, a statistically significant association was found between plasma Mg concentrations (basically in the highest quartile) and greater total cholesterol (*p* < 0.05) and LDL-cholesterol concentrations (*p* < 0.05). In conclusion, our results contribute to increasing the evidence gathered by numerous studies on the inverse association between hypomagnesemia and type-2 diabetes, as well as to the observation, previously reported in some studies, of a direct association with hypercholesterolemia. This paradoxical link should be deeply investigated in further studies.

## 1. Introduction

Magnesium is considered as an essential mineral for the functioning of the organism, participating in approximately 80% of known metabolic functions [1] and playing both structural and regulatory roles in the organism [1,2]. It has been estimated that it is the fourth most abundant cation in the whole body and the second at the intracellular level [3,4]. Magnesium is an essential co-factor in the enzymatic pathways involved with energy, protein and lipid metabolism and the modulation of glucose transport through the cellular membrane [5,6,7,8]. Thus, magnesium serves as an important link between transport systems and metabolism, and its concentration in the cytoplasm is regulated in a highly precise manner [9,10,11]. Despite the importance of magnesium in the organism, there are various studies that indicate that a considerable percentage of the population may have magnesium intake lower than the optimum [12,13,14]. Thus, it has been estimated that 40%–50% of adults do not achieve the average dietary intake (ADI) [14,15,16]. Although it is problematic to find good indicators of so-called magnesium deficiency in organisms [14,17,18,19,20], a number of studies have been undertaken to reveal the percentage of the population that presents hypomagnesemia and its negative repercussions on health [20,21,22,23]. Many studies carried out on various populations have generally supported the protective role that magnesium plays when faced with different human health problems [22,23,24,25,26]. Thus, it is no surprise to find recommendations of increasing the consumption of magnesium to improve health. The scientific evidence backing up that recommendation is based on various randomized clinical trials in which magnesium supplements have been shown to improve several of the parameters analyzed, including plasma lipids, glycemic control, blood pressure, metabolic syndrome, inflammatory markers and endothelial function [27,28,29,30,31,32,33], among others. However, there are also other intervention studies that have detected no such improvements [34,35,36,37]. Although intervention studies with Mg supplements and under controlled experimental conditions may provide a high level of scientific evidence, these studies also have limitations related with small sample size and special population characteristics, and so it is necessary to obtain additional evidence from other studies, even though they be of an observational nature. Among those observational studies, much research has been undertaken on whether there exists a relationship between magnesium contribution through dietary food and different cardiovascular risk factors, diabetes and cardiovascular diseases [38]. Thus, some studies have reported inverse associations between dietary magnesium intake and body mass index (BMI) or obesity [39,40], blood pressure [40,41], total cholesterol/LDL-cholesterol concentrations [42], and fasting glucose or type-2 diabetes [41,43,44], but the overall consistency for some of these factors is still low. Regarding the incidence of cardiovascular diseases, some studies have reported that high magnesium intake is associated with a lower risk of stroke, heart failure and total cardiovascular events [45,46], but less agreement has been observed for other cardiovascular events [38,47]. Given the undoubted limitations to assessing the amount of magnesium intake through dietary foods, due to several biases derived from the use of food frequency questionnaires, the use of magnesium intake/status biomarkers is preferred as a more objective measure. Although there are still no perfect magnesium intake/status biomarkers [15,47,48,49], plasma/serum magnesium concentrations are the most widely practiced and accepted method of determining magnesium status in epidemiological studies [15,17,19], having more advantages than urinary magnesium concentrations that are more dependent on kidney function [50].

Several prospective cohort studies have analyzed plasma magnesium concentrations and cardiovascular disease incidence and diabetes risk, and some of them reported inverse associations in a meta-analysis [51]. Although at the cross-sectional level numerous researchers have investigated associations between plasma magnesium concentrations and cardiovascular risk factors including plasma lipids, blood pressure, fasting glucose/diabetes, inflammation markers, among others, the consistency (except for type-2 diabetes), is still low [14,52,53,54,55,56,57,58]. Therefore, further studies are required on different populations in order to better understand those associations as well as the demographic and lifestyle factors associated with plasma magnesium concentrations. Hence, the objectives of our study were as follows: (1) to analyze plasma magnesium concentrations in a Mediterranean population and to investigate the influence of sex, age, urinary magnesium, tobacco smoking and adherence to the Mediterranean diet on those concentrations; and (2) to study the association between plasma magnesium concentrations and various cardiovascular risk phenotypes including body mass index (BMI), waist circumference, blood pressure, plasma lipid concentrations, fasting plasma glucose, type-2 diabetes, plasma creatinine, uric acid and aspartate aminotransferase in this population.

## 2. Materials and Methods

### 2.1. Study Design and Participants

We have carried out a cross-sectional analysis on 492 Caucasian subjects participating in the OBENUTIC-Mineral study. OBENUTIC stands for Obesity, Nutrition and Information and Communication Technologies and it is a case-control study conducted in the general population of the Valencia Region, Spain [59]. The ages of the participants in the OBENUTIC study were between 18 and 80 years old and included both men and women. Cases were obese subjects (body mass index (BMI) ≥ 30 kg/m^2^) and the controls were non-obese individuals (BMI < 30 kg/m^2^) recruited from the same region, without pairing for age and sex. The exclusion criteria were being pregnant or breast-feeding, invalidating physical or psychological diseases, cancer diagnosis, thyroid alterations, Cushing disease, suffering from some type of infectious/contagious disease, high alcohol intake or the consumption of other drugs. From participants in the OBENUTIC study, carried out over several years and with a greater sample size, a sub-set of 500 individuals recruited consecutively over 22 months was pre-selected for the OBENUTIC-Mineral sub-study. Of those, biological samples were obtained from 492 individuals in enough quantity to carry out the mineral determinations. In this particular study, focusing on measuring plasma magnesium concentrations as the main variable, 8 plasma samples with extreme values that deviate from other observations on data were detected, so indicating potential measurement errors or other types of incidence in the handling or conservation of the samples. These were considered as outliers, and the 8 individuals whose samples presented these outliers for magnesium concentrations were excluded from the study. Hence, in this work, 484 participants were finally included in the statistical analyses (160 men and 324 women), aged between 18 and 80 years old. Participants were apparently healthy individuals recruited through advertisements in shopping malls, housewives’ associations, cultural associations and other types of groups from the general population, public and private institutions, educational centers, some primary health care centers and home contacts. The study was undertaken at the Department of Preventive Medicine and Public Health, School of Medicine at the University of Valencia, Valencia. Participants provided written informed consent and study protocol and procedures were approved according to the ethical standards of the Helsinki Declaration and by the Human Research Ethics Committee of the University of Valencia, Valencia (reference number: H1488282121722).

### 2.2. Demographic, Anthropometric, Biochemical, Clinical and Lifestyle Variables

Socio-demographic, clinical variables (personal and family history of disease), medication use and lifestyle variables (smoking, physical activity, etc.) were obtained through a standardized questionnaire previously used in our studies [60]. Participants were measured for height with a standard stadiometer incorporated into the scales (SECA Mod 220, Seca Deutschland Gmbh and Co. Kg., Hamburg, Germany). Weight was determined with calibrated scales (TANITA-BC-420-S, Tanita UK Ltd., Middlesex, UK) [59]. Body Mass Index (BMI) was calculated as the weight in kilograms divided by the square of height in meters. Obesity was defined as having a BMI ≥ 30kg/m^2^. Waist circumference was measured midway between the lowest rib and the iliac crest using an anthropometric tape. Waist circumference was considered high when ≥ 102 cm in men or ≥ 88 cm in women. Systolic and diastolic blood pressures were obtained using an automatic sphygmomanometer (Omron HEM-705CP, OMRON Healthcare Europe B.V., Hoofddorp, The Netherlands). Hypertension was defined as systolic blood pressure ≥ 140 mmHg or diastolic blood pressure ≥ 90 mmHg or if subjects were receiving antihypertensive medication. Blood samples were collected after a 12-hour overnight fast. Plasma samples were obtained by centrifugation and the standard biochemical analyses were carried out the same day. In addition, plasma samples were stored at −80 °C for later analyses (i.e., magnesium determinations). Fasting plasma glucose, total cholesterol, HDL-cholesterol and triglyceride concentrations were measured using standard enzymatic methods as previously described [59] and LDL-cholesterol was estimated by the Friedewald equation (Olympus AU5400, Beckman Coulter, California, USA). Plasma creatinine was measured by the Jaffé method; uric acid was measured by the uricase method; and aspartate aminotransferase was measured by a standard method implemented on a Roche Diagnostics (Basel, Switzerland) multi-autoanalyzer in the same certified clinical laboratory. Diabetes was defined as having a fasting glucose ≥ 126 mg/dL or drug treatment for diabetes). Hypercholesterolemia was defined as LDL ≥ 160 mg/dL or taking lipid-lowering drugs. In addition, a first voided sample of urine, obtained the same day of the blood sample, was collected and stored at −80 °C for later analyses.

The level of adherence to the Mediterranean diet was measured by a validated 14-item questionnaire [61]. Registered dietitians completed the validated 14-item Mediterranean diet adherence screener (Supplemental Table S1) in a face-to-face interview with the participant. Each item was scored 0 or 1. The value of adherence to the Mediterranean diet was calculated as the summation of the 14 questions. A total score of 14 points indicates maximum adherence to the Mediterranean diet, whereas zero points indicates no adherence. For the total Mediterranean diet score, the greater the score, the greater the adherence. This variable was used as continuous and was also categorized for the statistical analyses. Subjects were classified as having low Mediterranean diet adherence (less than 9 points) or high adherence (≥ 9 points), based on the population mean of a previous study in our Mediterranean population [61]. For physical activity, sedentary status was defined as the auto-reported walking of less than 20 min a day. According to the World Health Organization, a current smoker was considered as someone who smokes any tobacco product at least once a day. Non-smokers included never and former smokers) [59].

### 2.3. Magnesium Determinations

Plasma magnesium was determined using an inductively coupled plasma mass spectrometer (ICP-MS) model 7500 supplied by Agilent Technologies (Agilent, Tokyo, Japan), using a carrier gas flow of 1.03 L/min, collision gas (He) flow of 4.3 mL/min, RF power of 1550 W and energy discrimination of 3 V, as previously reported [62]. All lenses were optimized daily. All materials used in the analyses were previously cleaned with supra-pure nitric acid and ultra-pure water (18.2 Ω) obtained using a Milli Q system. Samples and the certified reference material (Seronorm Trace Elements Serum L-1, Billingstad, Norway) were prepared by attack with nitric acid and hydrogen peroxide (supra-pure quality, Merck, Germany) in a microwave digester (Milestone, Sorisole, Italy). When the samples had been digested, the extracts were collected and made up to a final volume of 10 mL with-ultra pure water for subsequent analysis. The calibration curve was prepared following the Ga addition technique (adding 0.04 mg/L) as an internal standard, using stock solutions of 1000 mg/L of magnesium (Merck, Germany). The accuracy of the method was evaluated by analysis of the certified reference material, obtaining the value of 8.2 ± 0.2 μg/L (certified value 7.8–8.8 μg/L), and by recovery studies, obtaining a recovery of 93%. The mean of five separate determinations was used. For assessing the magnesium in urine, first morning urine samples were collected as previously indicated. Urine was not collected over 24 hours as recommended for the preferred method of urinary magnesium excretion determination [63], given that the urine samples in our study were already obtained and stored. Urine samples were prepared with an acidic solution containing 1% NHO3 and 0.5% HCl. The determination of magnesium total content in urine samples was also performed by means of an ICP-MS instrument provided of a He collision cell. Calibration curves were prepared using Ga as an internal standard and by the dilution of stock solutions of 1.000 mg/L in 1% HNO3 (all reagents were supra-pure from Merck, Germany). The accuracy of this method for urine was evaluated by comparison with certified reference materials Seronorm™ Trace Elements Urine (Billingstad, Norway) and by recovery studies of spiked samples with multi-element standards. The calculated recovery was between 95% and 105% in all cases. The mean of separate determinations was used.

### 2.4. Statistical Analysis

Firstly, it was checked whether the continuous variables followed normal distribution to detect any outliers. For plasma magnesium concentrations, 8 outliers were detected in the 492 initially included participants. So that those values would not influence the later statistical analyses, it was decided to exclude those 8 individuals with those extreme determinations, and the analyses were then undertaken on the remaining 484 participants. The triglyceride variable in plasma was logarithmically transformed for statistical testing. Descriptive statistics were used to analyze the variables for socio-demographic, clinical and anthropometric data. Chi-squared tests were used to compare proportions. The t-test and ANOVA were used to compare crude means of continuous variables. Multivariate adjustments for comparisons of continuous variables were carried out by generalized linear models for continuous variables and multivariate logistic regression used for dichotomous variables. In addition to its use as a continuous variable, plasma magnesium was also used as a categorical variable. Four groups were created considering the population quartiles (Q) as follows: Q1 from 0.47 to 0.71 (mmol/L), Q2 from 0.72 to 0.77 (mmol/L), Q3 from 0.78 to 0.83 (mmol/L) and Q4 from 0.84 to 1.13 (mmol/L). Overall quartiles were used rather than sex-specific quartiles because no differences between sexes in plasma magnesium concentrations were detected. For the quartile analyses, p values for trend were calculated using the quartile median values. The age variable used as continuous was also categorized, taking the tertiles of the population into account. Multivariable logistic regression methods were used to estimate the odds ratios (OR) and the 95% confidence intervals (CI) associated with the corresponding risks as indicated. For both continuous and dichotomous variables, several models were used for estimating the sequential adjustment as follows: Model 1, unadjusted model; Model 2, model adjusted for age, sex and obesity; Model 3, model adjusted for age, sex, obesity, diabetes and medications (lipid-lowering drugs, antidiabetic drugs or antihypertensive drugs). Additional adjustments (also including the tests for interaction terms with sex or with obesity status in the corresponding regression models) were carried out for specific analysis and were indicated in the text. Analyses were mainly undertaken on the whole sample studied and stratified by sex or obesity when indicated. For urine magnesium analysis, 4 outliers were detected and removed from the presented statistical results. Even after the removal of the outliers, urine magnesium did not reach a normal distribution and a square root transformation for this variable was carried out. Data were analyzed using SPSS Statistics for Windows Ver. 26 (IBM Corp., Armonk, NY, USA). All tests were two-tailed and *P*-values <0.05 were considered statistically significant.

## 3. Results

### 3.1. Characteristics of the Population and Plasma and Urine Magnesium Concentrations

Table 1 shows the general characteristics of the studied population. The mean age was 46.28 ± 13.73 years, with no statistically significant differences between men and women (*p* = 0.520). The prevalence of obesity cases was 32%, being slightly higher in men than in women (*p* = 0.021). The prevalence of type-2 diabetes in this general population was low (5.41%), being higher in men (8.50%) than in women (3.88%); *p* = 0.039. Mean plasma magnesium in this population was 0.77 ± 0.08 mmol/L and no statistically significant differences between men and women were observed. Hypomagnesemia, defined as having plasma magnesium concentrations < 0.70 mmol/L [64], was 18.6% in the whole population. No statistically significant differences in the prevalence of hypomagnesemia between men (17.5%) and women (19.1%) were detected (*p* = 0.663). Under normal conditions, plasma magnesium levels range from 0.66 to 1.05 mmol/L and are influenced by the balance between intestinal absorption and renal excretion [65]. In this sample, no subject was detected that presented hypermagnesemia. In addition to plasma magnesium, urinary magnesium was analyzed in a first morning urine sample and expressed it as mmol/L. Urine was not collected over 24 hours. Therefore, urinary magnesium in 24-hour urine was not calculated, even though this marker is preferred [63]. Thus, urine magnesium concentration was only used as a descriptive marker in this work and its association with cardiovascular risk factors was not analyzed. There are several works showing a good correlation between magnesium concentrations in the early morning urine sample and magnesium in the 24-hour urine collection method [66,67]. Some authors, however, have indicated that the early morning urine sample and the 24-hour urine collection cannot be used interchangeably in the evaluation of urinary magnesium excretion, as a good correlation does not translate into an agreement between the two measurements [68]. In the whole population, the mean urine magnesium concentration was 3.95 ± 2.17 mmol/L. Statistically significant differences were found between men and women (4.24 ± 2.13 mmol/L versus 3.81 ± 2.18 mmol/L, respectively; *p* = 0.045). This difference remained statistically significant even after multivariate adjustment for age, obesity, diabetes and medications (*p* = 0.030). Likewise, urinary magnesium concentrations were statistically different by age (*p* < 0.001), being lower in the older age group (Supplemental Figure S1). The correlation between plasma and urine magnesium (square root transformation for normality) concentrations was analyzed in the whole population and a direct statistically significant association (r = 0.150; *p* = 0.001) was found. As expected, despite being statistically significant, the magnitude of this correlation is low.

### 3.2. Association between Plasma Magnesium Concentrations and Demographic and Lifestyle Variables

Table 2 shows mean plasma magnesium concentrations depending on sex, age groups, tobacco smoking, sedentary lifestyle and adherence to Mediterranean diet. *P*-values are presented unadjusted and sequentially adjusted for the indicated potential confounders. In Model 3, men tended to have higher plasma magnesium concentrations than women, but differences did not reach statistical significance (*p* for Model 3: 0.059). No significant differences were found in plasma magnesium concentrations by age group. Likewise, plasma magnesium concentrations did not differ by smoking status, sedentarism, or adherence to the Mediterranean diet (*p* > 0.05 for all). In Table 2, adherence to the Mediterranean diet was considered as a categorical variable (high adherence versus low adherence based on the previously established cut-off point of nine points). Even after adjustment for sex, age, obesity, diabetes and medications, mean plasma magnesium values were similar in subjects with a high adherence (0.77 ± 0.08 mmol/L in the low adherence level versus 0.77 ± 0.08 mmol/L in the high adherence; *p* = 0.665). Likewise, when adherence to Mediterranean diet was considered as a continuous variable, no statistically significant association with plasma magnesium levels was detected (*p* = 0.0728).

However, when the specific foods of the Mediterranean diet were analyzed (Supplemental Table S2), some statistically significant differences were detected. Interestingly, two of the items related to olive oil consumption presented statistically significant associations with plasma magnesium concentrations. Individuals indicating the use of olive oil as the principal source of fat for cooking had a mean concentration of plasma magnesium of 0.77 ± 0.08 mmol/L versus 0.74 ± 0.07 mmol/L in subjects not using olive oil as the main fat. Likewise, an affirmative response to the second item (“use of 4 or more tablespoons of olive oil/d”) was also associated with higher plasma magnesium concentrations 0.78 ± 0,08 mmol/L versus 0.75 ± 0.08 mmol/L, in non-consumers; *p =* 0.012. These results remain statistically significant even after additional multivariate adjustment for age, sex, obesity, diabetes and medications. No statistically significant associations were detected for the other items.

### 3.3. Associations between Plasma Magnesium and Cardiovascular Risk Factors

The association between plasma magnesium concentrations and cardiovascular risk factors were then analyzed. Table 3 shows the results for anthropometric and clinical variables. No statistically significant associations were observed for waist circumference, or obesity categories. Conversely, statistically significant associations were identified with type-2 diabetes and hypercholesterolemia. Plasma magnesium concentrations were lower in type-2 diabetic subjects in comparison with non-diabetic subjects (0.73 ± 0.13 mmol/L vs. 0.77 ± 0.08 mmol/L; *p* = 0.006 in a model adjusted for sex, age, obesity, diabetes and medications). Concerning hypercholesterolemia, a statistically significant association was also detected, but plasma magnesium concentrations were higher in hypercholesteremic subjects compared to non-hypercholesteremic (0.79 ± 0.09 mmol/L versus 0.76 ± 0.07 mmol/L; *p* = 0.001 in a multivariate model adjusted for sex, age, obesity, diabetes and medications).

The relationship between hypomagnesemia (plasma magnesium < 0.70 mmol/L) and prevalence of diabetes (Table 4) was then examined. The prevalence of type-2 diabetes was 11.24% in subjects with hypomagnesemia and 3.11% in subjects with normomagnesemia. Hypomagnesemia was higher in type-2 diabetic subjects in comparison to non-diabetics (40.0% versus 17.4%; *p* = 0.008, respectively). In the multivariate adjusted Model 3, the odds of being type-2 diabetic in subjects with hypomagnesemia was high, OR: 3.36 (95% CI: 1.26–8.96); *p* = 0.016.

However, hypomagnesemia was not statistically related to hypercholesterolemia. In the multivariate logistic regression model adjusted for sex, age, obesity, diabetes and medications, the odds for hypercholesterolemia did not reach statistical significance (OR: 1.38; 95%CI: 0.81–2.35; *p* = 0.233). To better understand the dose–response relationship between plasma magnesium and cholesterol as well as with the other cardiovascular risk factors, quartiles of plasma magnesium were created and the association between these quartiles and the corresponding cardiovascular risk variables was studied (Table 5).

Plasma magnesium was not statistically associated with blood pressure, HDL-cholesterol, fasting triglycerides, uric acid or aspartate aminotransferase in this sample. In Model 3, however, statistically significant results for total cholesterol (*p* = 0.010 in Model 3) were observed as well as for LDL-cholesterol (*p* = 0.002 in Model 3). According to these results, higher plasma magnesium concentrations were directly related to higher total cholesterol and LDL-cholesterol, mainly in the fourth quartile. This result is contrary to the previously observed relationship with diabetes. Likewise, another statistically significant direct association was detected with creatinine levels (*p* = 0.032 in Model 3). As higher plasma creatinine is an indicator of altered renal function, additional adjustments were made for creatinine concentrations in Model 3 to better understand the association between plasma magnesium and total cholesterol and LDL-cholesterol. After this additional adjustment, both total cholesterol and LDL-cholesterol remained statistically associated with higher plasma magnesium levels.

When the influence of obesity on the magnesium effect on biochemical parameters was analyzed by estimating the statistical significance of the interaction term between obesity and magnesium in determining the corresponding cardiovascular risk factor, no statistically significant interaction terms were found for any of the variables analyzed in the multivariate adjusted models. The *p* values for the corresponding interaction terms were as follows: for fasting glucose: *p =* 0.467; for total cholesterol: *p =* 0.157; for LDL-cholesterol: *p =* 0.282; for HDL-cholesterol: *p =* 0.194; for triglycerides: *p =* 0.846; for creatinine: *p =* 0.549; for SBP: *p =* 0.592; and for diastolic blood pressure: *p =* 0.702 (not shown in tables). Likewise, heterogeneity per sex was analyzed for these parameters, but no statistically significant interaction term was observed. *P =* 0.412; for total cholesterol: *p =* 0.643; for LDL-cholesterol: *p =* 0.827; for HDL-cholesterol: *p =* 0.152; for triglycerides: *p =* 0.675; for creatinine: *p =* 0.105; for SBP: *p =* 0.789; and for diastolic blood pressure: *p =* 0.424 (not shown in tables).

Finally, we estimated the odds for hypercholesterolemia depending on the quartiles of plasma magnesium (Table 6). Although a statistically significant linear trend was detected between plasma magnesium and hypercholesterolemia OR: 1.41; 95%CI: 1.12–1.76 per standard deviation; *p =* 0.003 (in Model 3), the association was mainly propelled by the fourth quartile of plasma magnesium. Thus, subjects with a high plasma magnesium level have higher odds of having hypercholesterolemia than subjects in the first magnesium quartile: OR: 3.12; 95%CI: 1.66–5.85; *p* ˂ 0.001.

## 4. Discussion

In this study, the prevalence of hypomagnesemia was found to be 18.6% [64]. Although in the literature there is heterogeneity in defining the cut-off point for hypomagnesemia, among the different critical values mentioned—0.8 mmol/L, 0.75 mmol/L, and 0.7 mmol/L [69]—the critical value of 0.7 mmol/L used in our study has been widely reported in several studies [25,64,70,71,72,73] but not in others. It has been stated that hypomagnesemia ranges from 2.0% to 15% in healthy subjects, and between 14% and 48% in type-2 diabetic subjects [25,64,74,75,76,77]. In addition to the different population characteristics, this wide range in the reported prevalence most likely reveals the difference in the criteria for the definition of hypomagnesemia as well as the differences in the techniques and samples (plasma/serum) used in magnesium measurements. The prevalence of hypomagnesemia in our study was very high in type-2 diabetic subjects (40.0%) in comparison with non-diabetic subjects (17.4%). Despite the small prevalence of type-2 diabetes in this population (5.41%), the association with hypomagnesemia reached statistical significance and was a relevant finding in our study. Although this is not a new finding considering that since the 1940s, type-2 diabetes has been reported to be associated with hypomagnesemia [78], it is important as, even in this scarcely studied Mediterranean population, we have been able to replicate previous findings on the strong association between low plasma levels of magnesium and diabetes risk [52,54,64,65]. Despite this strong association, there is currently little interest in hypomagnesemia, and plasma magnesium is not generally measured in large cohorts from the general population or even in patients at risk of type-2 diabetes. The relative lack of symptoms of hypomagnesemia until plasma concentrations reach severely low levels as well as the relatively poor understanding of magnesium metabolism may have contributed to this relatively low awareness for hypomagnesemia. Recently, another study carried out in the south of Spain in patients with coronary heart disease recruited in the CORDIOPREV trial [53] also reported a strong cross-sectional association between serum magnesium concentration and the prevalence of type-2 diabetes. Moreover, in this study, serum magnesium was strongly and inversely associated with the Carotid Intima-Media Thickness, even after adjustment for diabetes. Despite the widespread clinical and epidemiological evidence of the association of hypomagnesemia and type-2 diabetes, it is not entirely clear if this association is the cause or a consequence. It has been reported that several drugs, including antidiabetic drugs and other drugs used for diabetes complications, are associated with low plasma magnesium [79,80,81] and can contribute to the hypomagnesemia observed in type-2 diabetic subjects. However, it has been estimated that only a minor part of hypomagnesemia can be explained by drug consumption [79]. Due to the cross-sectional nature of our study, more factors determining the low plasma magnesium in type-2 diabetic subjects could not be investigated. However, some cohort studies have prospectively analyzed the incidence of new cases of type-2 diabetes and have reported an inverse association with plasma magnesium concentrations [51,82,83,84], minimizing the cross-sectional bias. Thus, in a cohort of more than 12,000 nondiabetic subjects from the Atherosclerosis Risk in Communities Study during 6 years of follow-up [82], the authors detected an approximate two-fold statistically significant increase in incidence rate of type-2 diabetes comparing the highest to the lowest (categorized into 6 groups). Likewise, Guerrero-Romero at al. [83] analyzed the risk conferred by hypomagnesemia of new onset type-2 diabetes after 10 years of follow-up and estimated a relative risk (RR) of 2.5; (95%CI: 1.1–4.1). In a meta-analysis of the published cohort studies [51], the pooled RR for incidence of type-2 diabetes comparing the highest to the lowest category of magnesium concentrations was RR: 0.64; 95% CI: 0.50–0.81). Likewise, numerous cohort studies analyzing dietary magnesium intake also found a protective effect, though of less magnitude. Thus, in a meta-analysis including 25 cohorts [85] comprising more than 600,000 individuals (approx. 27,000 type-2 diabetics), the highest category of magnesium intake compared with the lowest, reduced the risk of type-2 diabetes across all the cohorts by 17%. Similarly, a meta-analysis of randomized controlled trials on the effects of magnesium supplementation on fasting glucose and insulin sensitivity [37] concluded that magnesium supplementation for more than 4 months significantly improved insulin resistance and fasting glucose in diabetic and in non-diabetic individuals. However, the mechanisms by which magnesium can improve insulin resistance and decrease diabetes risk are still far from understood [64].

Focusing on factors related to serum magnesium concentrations, no statistically significant differences were detected per sex, age, BMI, or smoking status in our population. These factors have been analyzed in various studies [15,53,55,63,77,86], obtaining different results depending on the population analyzed. Mataix et al. [15], in a study undertaken to identify factors influencing plasma magnesium levels in the general population of southern Spain, and in agreement with our results, did not observe any association between plasma levels of magnesium and sex, age, obesity, tobacco smoking, alcohol drinking, educational level or sedentarism. Also, in southern Spain, but analyzing subjects at high cardiovascular risk, Rodriguez-Ortiz at al. [53] detected statistically significant age differences, but no differences depending on sex, BMI or tobacco smoking. Conversely, Bertinato et al. [86], analyzing a representative population from Canada, described statistically significant differences in serum concentrations depending on the sex, age and BMI. Genetic factors [87,88] and/or dietary intake may modulate these associations and contribute to the differences among populations. Although there are several genetic polymorphisms in the transient receptor potential cation channel subfamily M member 6 (*TRPM6*) gene and in the ADP Ribosylation Factor Like GTPase 15 (*ARL15*) gene, among others [87,88] that have been related to magnesium levels, this analysis was not the focus of our study and their contribution in this populations remains to be investigated. Nevertheless, diet in this population at the dietary pattern level was analyzed. A validated questionnaire was used for measuring adherence to the Mediterranean diet pattern [61]. No statistically significant association was found between total adherence to the Mediterranean diet and plasma magnesium concentrations in this population. This observation was relatively expected because previous studies analyzing at the same time the correlation between total dietary magnesium intake, assessed by questionnaire, and plasma magnesium concentrations did not observe significant associations [14,64,82,89,90]. One limitation of our study is that the amount of magnesium provided by dietary foods was not measured and so this direct measure was not available to evaluate the association between total magnesium intake and plasma magnesium concentration. We focused on the Mediterranean diet pattern because the number of studies analyzing the association between the Mediterranean diet pattern and plasma magnesium concentrations is very scarce. Using a different instrument for measuring the adherence to the Mediterranean diet [91], the Mediterranean diet quality index (Med-DQI), instead of our validated 14-item score for adherence [61], Bahreini et al. [91] in Iran measured Mediterranean diet adherence and observed a significant association with serum magnesium concentrations in 102 patients. Higher adherence to the Mediterranean diet was associated with higher magnesium concentrations. In our study, despite not observing a significant association with total adherence to the Mediterranean diet, significant associations were found with two items of the score related to the olive oil consumption. Hence, in our study, subjects who consumed olive oil as the main fat for cooking and had more than four spoonfuls a day had higher plasma magnesium concentrations. As this is the first time that this association has been reported, we cannot assess its plausibility or say whether this was by chance. It is possibly due to an indirect association with other factors, as olive oil per se does not contribute any significant concentrations of magnesium. It is known that magnesium deficiency causes inflammatory and oxidative stress [1,2] and that, in turn, the anti-inflammatory and anti-oxidizing effect of virgin olive oil could mimic the protective effect of plasma magnesium, contributing to the observed higher levels. Until now, no studies have been published on whether virgin olive oil may be associated with higher levels of plasma magnesium and despite the results obtained, we are aware of the small number of individuals in our study, so it will be necessary to undertake these analyses in populations with a greater number of participants.

Finally, another unexpected finding in our study is the direct association found between plasma magnesium and total cholesterol and LDL-cholesterol concentrations. This finding seems contradictory taking into account that high LDL-cholesterol has been associated with higher cardiovascular disease in several studies [92] and, on the other hand, there are several studies showing that higher plasma magnesium concentrations is associated with lower cardiovascular disease risk [38,51,93]. The results of studies that have analyzed the influence of magnesium supplementation on plasma lipid concentrations have been contradictory [37,93]. Thus, Shahbah et al. [94], in a clinical trial on children, reported improvements in the atherogenic lipid profile (decreases in total cholesterol and LDL-cholesterol after intervention with magnesium supplements). Other intervention studies reached the same conclusion in adults; however, a meta-analysis of randomized controlled trials on the effects of magnesium supplementation on lipid profile [37] concluded that no decrease in total cholesterol or LDL-cholesterol was observed in the pooled analysis. Likewise, in observational studies, the results regarding total cholesterol and LDL-cholesterol are inconclusive [38]. Some studies showed a statistically significant inverse association between plasma magnesium and total or LDL-cholesterol [95,96], but other studies did not observe any significant association [38,55,57,97]. Moreover, there are numerous publications [53,56,57,98,99], that, in agreement with our results, found statistically significant direct associations between plasma magnesium concentrations and total and/or LDL-cholesterol levels, but they did not emphasize the findings. If confirmed in later studies, these findings may help to explain inconsistent results in some studies analyzing plasma magnesium and cardiovascular risk due to the concomitant increase in the atherogenic LDL-cholesterol, but more research must be done on the potential mechanisms involved.

## 5. Conclusions

In this study, undertaken on individuals coming from a general Mediterranean population from the East Mediterranean coast of Spain, no differences in plasma magnesium concentrations were found according to sex, age, obesity or tobacco smoking. Nor was any association found between magnesemia and total adherence to the Mediterranean diet, but significant associations with olive oil consumption were obtained, which require further investigation. No significant associations were found between plasma magnesium and blood pressure, plasma triglycerides, HDL-cholesterol, uric acid or aspartate aminotransferase. However, a statistically significant inverse association was confirmed between magnesemia and prevalence of type-2 diabetes, as previously reported in several studies reporting a protective role of plasma magnesium in this disease. Conversely, in agreement with some other cross-sectional studies, a direct association was detected between higher plasma magnesium and higher total cholesterol and LDL-cholesterol. This association is mainly observed in the highest quartile of magnesium concentrations. This result seems contradictory with studies that indicate a strong inverse association of plasma magnesium concentrations with lower cardiovascular risk and, therefore, more specific investigation in other studies is required.

## Figures and Tables

**Table 1:** Demographic, anthropometric, clinical and biochemical characteristics of the participants by sex.

	Total (*n* = 484)	Men (*n* = 160)	Women (*n* = 324)	*p*
Age (years)	46.28 ± 13.73	45.69 ± 14.75	46.58 ± 13.21	0.520
BMI (Kg/m^2^)	27.87 ± 5.44	29.17 ± 4.92	27.23 ± 5.58	<0.001
SBP (mm Hg)	124.81 ± 17.32	132.70 ± 15.88	120.90 ± 16.67	<0.001
DBP (mm Hg)	78.52 ± 10.87	82.38 ± 12.11	76.60 ± 9.66	<0.001
Total cholesterol (mg/dL)	211.94 ± 40.43	204.78 ± 38.86	215.47 ± 40.78	0.006
LDL-cholesterol (mg/dL)	137.82 ± 32.71	137.22 ± 32.21	138.10 ± 32.99	0.781
HDL-cholesterol (mg/dL)	59.65 ± 14.13	50.89 ± 11.03	63.94 ± 13.50	<0.001
Triglycerides (mg/dL)	108.68 ± 58.15	122.55 ± 66.79	101.85 ± 52.16	<0.001
Fasting glucose (mg/dL)	94.91 ± 19.57	99.07 ± 23.08	92.87 ± 17.25	0.003
Creatinine, mg/dL	0.76 ± 0.18	0.94 ± 0.19	0.10 ± 0.01	<0.001
Uric acid, mg/dL	5.31 ± 1.42	1.24 ± 0.10	1.16 ± 0.06	<0.001
Aspartate aminotransferase, U/L	25.31 ± 10.44	29.77 ± 1.13	23.11 ± 7.00	<0.001
Urine magnesium, mmol/L	3.95 ± 2.17	4.24 ± 2.13	3.81 ± 2.18	0.045
Plasma magnesium, mmol/L	0.77 ± 0.08	0.78 ± 0.07	0.77 ± 0.08	0.106
Hypomagnesemia %	18.6	17.5	19.1	0.663
Obesity cases %	32.0	39.6	28.2	0.012
Type 2 diabetes: %	5.41	8.50	3.88	0.039
Hypercholesterolemia %	35.7	39.9	33.6	0.186
Hypertension %	31.9	48.4	23.6	<0.001
Current smokers: %	20.1	16.2	21.9	0.118
Antidiabetic drugs %	3.25	4.67	2.57	0.235
Hypolipidemic drugs %	14.5	19.3	12.1	0.040
Antihypertensive drugs %	16.9	28.7	11.2	<0.001

Values are mean ± SE for continuous variables and % for categorical variables. BMI indicates body mass index; SBP indicates systolic blood pressure, DBP indicates diastolic blood pressure; *p*: *p*-value for the comparisons (means or %) between men and women; hypomagnesemia: Plasma magnesium < 0.70 mmol/L; obesity: BMI ≥ 30 kg/m^2^; type 2 diabetes: Antidiabetic drug or glucose ≥ 126 mg/dL; hypercholesterolemia: Hypolipidemic drugs or LDL-cholesterol ≥ 160 mg/dL. hypertension: antihypertensive drug or PAS ≥ 140 mmHg or PAD ≥ 90 mmHg.

**Table 2:** Associations between plasma magnesium concentrations and socio-demographic and lifestyle variables in the whole population.

Variables	Categories	Magnesium (mmol/L)	*P* ^1^	*P* ^2^	*P* ^3^
Sex	Men	0.78 ± 0.07	0.128	0.112	0.059
	Women	0.77 ± 0.08			
Age	18–42 years	0.77 ± 0.07	0.780	0.633	0.360
	43–54 years	0.77 ± 0.08			
	55–80 years	0.77 ± 0.09			
Smoking	Non-smokers	0.77 ± 0.08	0.591	0.645	0.510
	Current smokers	0.77 ± 0.08			
Sedentarism	No sedentarism	0.76 ± 0.08	0.153	0.183	0.131
	Sedentarism	0.78 ± 0.08			
Adherence to Mediterranean diet	Low-adherence	0.77 ± 0.08	0.643	0.788	0.644
	High-adherence	0.77 ± 0.08			

Variables are expressed as mean (± SD); *p*: *P*-value for the comparisons (means) between levels of the different categories; ^1^: *P*-value for the Model 1 (unadjusted); ^2^: *P*-value for Model 2 (adjusted for sex, age and obesity); ^3^: *P*-value for Model 3 (Model 2 additionally adjusted for diabetes and medications). A total of 484 subjects were analyzed for the comparisons.

**Table 3:** Associations between plasma magnesium concentrations and anthropometric and clinical variables in the whole population.

Variables	Categories	Magnesium (mmol/L)	*P* ^1^	*P* ^2^	*P* ^3^
Waist circumference, cm	Low waist	0.77 ± 0.08	0.677	0.484	0.627
	High waist	0.77 ± 0.08			
BMI, kg/m^2^	Normal weight	0.76 ± 0.07	0.300	0.249	0.326
	Overweight	0.78 ± 0.08			
	Obesity	0.77 ± 0.09			
Smoking	Non-smokers	0.77 ± 0.08	0.591	0.645	0.510
	Current smokers	0.77 ± 0.08			
Hypertension	No hypertension	0.77 ± 0.08	0.319	0.593	0.507
	Hypertension	0.78 ± 0.08			
Type-2 diabetes	No diabetes	0.77 ± 0.08	0.116	0.003	0.009
	Diabetes	0.73 ± 0.13			
Hypercholesterolemia	No hypercholesterolemia	0.76 ± 0.07	0.002	0.004	0.001
	Hypercholesterolemia	0.79 ± 0.09			

Variables are expressed as mean (± SD); *p*: *P*-value for the comparisons (means) between categories of the different variables analyzed; ^1^: *P*-value for Model 1 (unadjusted): ^2^: *P*-value for Model 2 (adjusted for sex, age and obesity); ^3^: *P*-value for Model 3 (Model 2 additionally adjusted for diabetes and medication where appropriate). Waist circumference was considered high when ≥ 102 cm in men or ≥ 88 cm in women. Normal weight: BMI < 25 kg/m^2^; overweight: BMI ≥ 25 Kg/m^2^ and BMI<30 kg/m^2^; obesity: BMI ≥ 30 kg/m^2^; type-2 diabetes: use of antidiabetic drugs and/or fasting glucose ≥ 126 mg/dl; hypercholesterolemia: using of lipid lowering drugs and/or plasma LDL-cholesterol ≥ 160 mg/dl. hypertension: antihypertensive drugs or PAS ≥ 140 mmHg or PAD ≥ 90 mmHg.

**Table 4:** Association between hypomagnesemia^1^ and prevalence of type-2 diabetes.

Mg	Non T2D (%)	T2D (%)	Model 1 OR (95% CI) *P* ^2^	Model 2 OR (95% CI) *P* ^3^	Model 3 OR (95% CI) *P* ^4^
Normomagnesemia	96.09	3.91	1.00 (reference)	1.00 (reference)	1.00 (reference)
Hypomagnesemia	88.76	11.24	3.11 (1.35–7.19) 0.008	3.01 (1.22–7.42) 0.017	3.36 (1.26–8.96) 0.016

^1^: Hypomagnesemia was considered when plasma magnesium < 0.70 mmol/L. OR: odds ratio; CI: confidence interval. T2D: type-2 diabetes. Multivariate logistic regression analysis: ^2^: Model 1 (OR, CI and *p*-value for unadjusted model); ^3^: Model 2 (OR, CI and *p*-value for Model 1 adjusted for sex, age and obesity); ^4^: Model 3 (OR, CI and *p*-value for Model 2 adjusted for sex, age, obesity and medications).

**Table 5:** Association between the plasma magnesium quartiles (Q) and plasma lipids, fasting glucose, creatinine, uric acid and aspartate aminotransferase in the whole population.

	Q1 (0.47–0.71 mmol/L)	Q2 (0.72–0.77 mmol/L)	Q3 (0.78–0.83 mmol/L)	Q4 (0.84–1.13 mmol/L)	*P* ^1^	*P* ^2^	*P* ^3^
*n*	121	122	121	120			
SBP, mmHg	124.06 ± 17.68	123.22 ± 17.75	124.79 ± 17.87	127.22 ± 15.83	0.314	0.692	0.656
DBP, mmHg	77.25 ± 11.97	78.81 ± 10.60	78.49 ± 11.10	79.54 ± 9.70	0.428	0.505	0.331
Total-cholesterol, mg/dL	206.85 ± 35.63	210.20 ± 39.25	208.88 ± 41.09	221.91 ± 44.09	0.017	0.010	0.010
LDL-cholesterol, mg/dL	132.60 ± 28.89	135.51 ± 32.10	136.17 ± 32.39	147.05 ± 35.66	0.003	0.004	0.002
HDL-cholesterol, mg/dL	59.26 ± 14.80	60.48 ± 14.11	59.53 ± 14.26	59.32 ± 13.46	0.901	0.781	0.933
Triglycerides, mg/dL	110.36 ± 56.26	106.12 ± 52.57	105.53 ± 62.68	112.79 ± 61.00	0.570	0.800	0.959
Glucose, mg/dL	99.93 ± 29.66	91.66 ± 13.86	94.49 ± 13.50	93.62 ± 15.80	0.007	0.003	0.223
Creatinine, mg/dL	0.74 ± 0.14	0.736 ± 0.17	0.80 ± 0.24	0.770±0.16	0.030	0.049	0.032
Uric acid, mg/dL	5.22 ± 1.42	5.24 ± 1.38	5.34 ± 1.58	5.43 ± 1.32	0.625	0.986	0.982
Aspartate aminotransferase, U/L	24.49 ± 9.92	25.26±11.08	25.57 ± 10.13	25.89 ± 10.64	0.801	0.944	0.873

Values are mean±SD for continuous variables. SBP indicates systolic blood pressure. DBP indicates diastolic blood pressure; *p*: *p*-value for trend across quartiles for the comparisons (means); ^1^: *p*-value for Model 1 (unadjusted): ^2^: *p*-value for Model 2 (adjusted for sex, age and obesity); ^3^: *p*-value for Model 3 (Model 2 additionally adjusted for diabetes and medications (lipid-lowering, antihypertensive drugs, antidiabetic drugs) when appropriate).

**Table 6:** Association between quartiles (Q) of plasma magnesium levels and the risk of hypercholesterolemia.

Q	No Hypercho-Lesterolemia (%)	Hyper-Choleste-Rolemia (%)	Model 1 OR (95% CI) *p* ^1^	Model 2 OR (95% CI) *p* ^2^	Model 3 OR (95% CI) *p* ^3^
Q1	72.03	27.97	1.00 (reference)	1.00 (reference)	1.00 (reference)
Q2	67.23	32.77	1.26 (0,72–2.19) 0.421	1.50 (0,80–2.80) 0.204	1.62 (0,85–3.10) 0.143
Q3	68.07	31.93	1.21 (0.69–2.11) 0.505	1.27 (0.68–2.39) 0.46	1.44 (0.75–2.77) 0.271
Q4	50.00	50.00	2.58 (1.50–4.42) 0.001	2.80 (1.52–5.16) 0.001	3.12 (1.66–5.85) ˂0.001
Linear effect ^4^			1.36 (1.12–1.65) 0.002	1.34 (1.08–1.66) 0.008	1.41 (1.12–1.76) 0.003

Q1: 0.47–0.71 mmol/L; Q2: 0.72–0.77 mmol/L; Q3: 0.78–0.83 mmol/L; Q4: 0.84–1.13 mmol/L. OR: odds ratio. CI: confidence interval. Multivariate logistic regression analysis: Model 1: unadjusted model; Model 2: adjusted for sex, age and obesity; Model 3: adjusted for sex, age, obesity, diabetes and medication. ^4^: For the estimation of the additive effect of plasma magnesium, the variable was considered as continuous and was first standardized. Thus, the OR expressed the increase in the risk per standard deviation of plasma magnesium concentrations. *p* ^1,2,3^: *p*-value obtained for the plasma magnesium variable in the corresponding multivariate logistic regression models.

## Supplementary Materials

The following are available online at https://www.mdpi.com/2072-6643/12/4/1018/s1: Supplemental Table S1: Quantitative 14-item questionnaire for Adherence to Mediterranean diet; Supplemental Figure S1: Urinary magnesium concentration depending on the age group in the whole population; Supplemental Table S2: Mediterranean diet adherence components and plasma magnesium concentrations depending on the item analyzed.
