# Supplementary material for: Influence of Demographic and Lifestyle Variables on Plasma Magnesium Concentrations and Their Associations with Cardiovascular Risk Factors in a Mediterranean Population
Source: Nutrients. 2020 Apr 8;12(4):1018. doi: 10.3390/nu12041018 (PMC7230301; doi:10.3390/nu12041018)
Supplement: Supplementary file 1 [file nutrients-12-01018-s001.pdf]

ONLINE SUPPORTING MATERIAL

Influence of demographic and lifestyle variables on plasma magnesium concentrations and their associations with cardiovascular risk factors in a Mediterranean population

Rocío Barragán, Juan Llopis, Olga Portolés, José V. Sorlí, Oscar Coltell, Lorenzo Rivas-García, Eva M. Asensio, Carolina Ortega-Azorín, Dolores Corella and Cristina Sánchez-González

Supplemental Table 1..2
Supplemental Figure 1 3
Supplemental Table 2..4

Supplemental Table 1. Quantitative 14-item questionnaire for Adherence to Mediterranean diet.

	Food items and frequency of consumption	Criteria for 1 point[1]
1	Do you use olive oil as main culinary fat?	Yes
2	How much olive oil do you consume per day (including that used in frying, meals eaten away from home, salads, etc.)? (1tablespoon = 13.5g)	≥ 4 (tablespoon)
3	How many serves of vegetables do you eat a day? How many servings of vegetables do you consume per day? (1 serving = 200g	≥ 2
4	How many fruit units (including natural fruit juices) do you consume per day?	≥ 3
5	How many servings of red meat, hamburger or meat products (ham, sausage, etc.) do you consume per week? (1 serving = 100–150g)	< 1
6	How many servings of butter, margarine, or cream do you consume per week? (1 serving = 12g)	< 1
7	How many sweetened beverages (soft drinks, cola, bitter, juices without added sugars) do you drink per week?	< 1
8	How much wine do you drink per week? (1 glass = 125 ml)	≥ 7 glasses
9	How many servings of legumes do you consume per week? (1 serving = 150g)	≥ 3
10	How many servings of fish or shellfish do you consume per week? (1 serving = 100-150g of fish or 4-5 units or 200g of shellfish)	≥ 3
11	How many times per week do you consume pastries, such as cookies, custard, sweets or cakes?	< 2
12	How many servings of nuts (including peanuts) do you consume per week? (1 serving = 30g)	≥ 3
13	Do you preferentially consume chicken, turkey, or rabbit meat instead of veal, pork, hamburger, or sausage?	Yes
14	How many times per week do you consume vegetables, pasta, rice, or other dishes seasoned with sofrito (sauce made with tomato and onion, leek, or garlic and simmered with olive oil)?	≥ 2

[1] '0' points if these criteria are not met.

Supplemental Figure 1. Urinary magnesium concentration depending on the age group in the whole population. Three age groups were created based on the population tertiles. Urinary magnesium concentrations were square root transformed for normality. Three models were fitted. Model 1: unadjusted model (P^1); Model 2: adjusted for sex, age and obesity (P^2) and Model 3: (P^3) additionally adjusted for diabetes and medications.

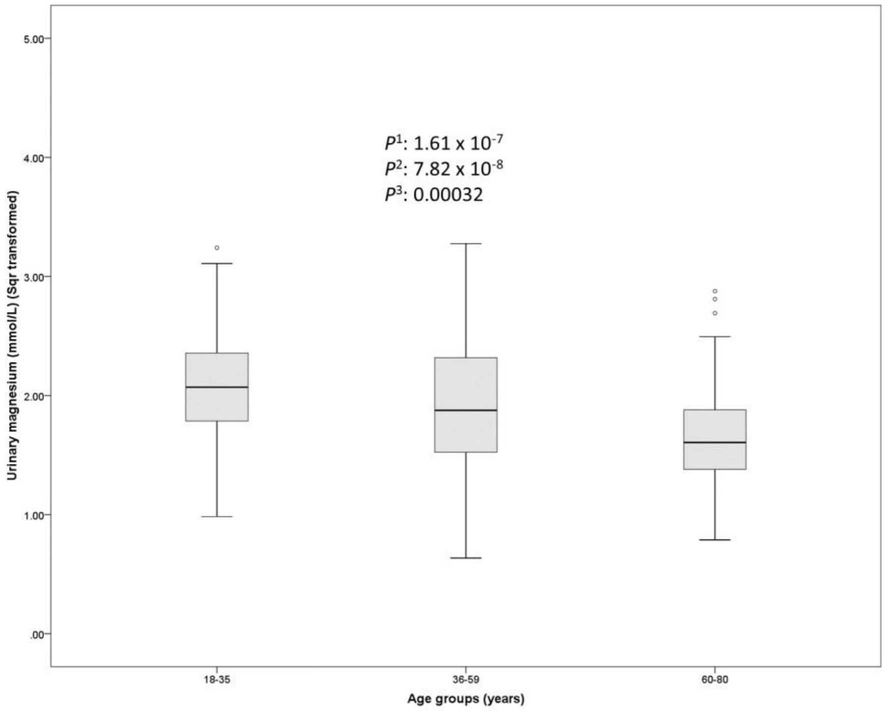

Supplemental Table 2. Mediterranean diet adherence components and plasma magnesium concentrations depending on the item analyzed in the whole population.

Item	%	Plasma Magnesium (mmol/L) (n=484)	P^1	P^2	P^3
**1. Do you use olive oil as the principal source of fat for cooking?**					
0 point	7.1	0.74 ± 0.07	**0.014**	**0.013**	**0.003**
1 point	92.9	0.77 ± 0.08			
**2. How much olive oil do you consume per day**					
0 point	25.7	0.75 ± 0.08	**0.012**	**0.020**	**0.023**
1 point	74.3	0.78 ± 0.08			
**3. How many serves of vegetables do you eat a day?**					
0 point	30.6	0.77 ± 0.08	0.538	0.589	0.684
1 point	69.4	0.77 ± 0.08			
**4. How many fruit units (including natural fruit juices) do you consume per day?**					
0 point	52	0.77 ± 0.08	0.873	0.738	0.665
1 point	48	0.77 ± 0.08			
**5. How many servings of red meat, hamburger or meat products do you consume per week?**					
0 point	27.2	0.77 ± 0.08	0.537	0.584	0.598
1 point	72.8	0.77 ± 0.08			
**6. How many servings of butter, margarine, or cream do you consume per week?**					
0 point	23.4	0.78 ± 0.09	0.467	0.567	0.502
1 point	76.6	0.77 ± 0.08			
**7. How many sweetened beverages (soft drinks, cola, bitter, juices without added sugars) do you drink per week?**					
0 point	28.1	0.77 ± 0.08	0.324	0.341	0.272
1 point	71.9	0.77 ± 0.08			
**8. How much wine do you drink per week?**					
0 point	86.2	0.77 ± 0.08	0.825	0.633	0.703
1 point	13.8	0.77 ± 0.08			
**9. How many servings of legumes do you consume per week?**					
0 point	78.1	0.77 ± 0.08	0.506	0.391	0.698
1 point	21.9	0.77 ± 0.09			
**10. How many servings of fish or shellfish do you consume per week?**					
0 point	53.3	0.77 ± 0.08	0.445	0.403	0.498
1 point	46.7	0.77 ± 0.08			
**11. How many times per week do you consume pastries, such as cookies, custard, sweets or cakes?**					
0 point	40.8	0.77 ± 0.08	0.940	0.766	0.646
1 point	59.2	0.77 ± 0.08			

**12. How many servings of nuts (including peanuts) do you consume per week?**					
0 point	55.4	0.77 ± 0.08	0.376	0.627	0.768
1 point	44.6	0.78 ± 0.08			
**13. Do you preferentially consume chicken, turkey, or rabbit meat instead of veal, pork, hamburger, or sausage?**					
0 point	19.2	0.77 ± 0.08	0.835	0.945	0.971
1 point	80.8	0.78 ± 0.08			
**14. How many times per week do you consume vegetables, pasta, rice, or other dishes seasoned with sofrito)?**					
0 point	26.6	0.77 ± 0.08	0.994	0.842	0.892
1 point	73.4	0.78 ± 0.08			

Variables are expressed as mean (± SD). p: *P-value* for the comparisons (means) between levels of the different categories. [1]: *P-value* for the model 1 (unadjusted). [2]: *P-value* for model 2 (adjusted for sex, age and obesity). [3]: *P-value* for model 3 (model 2 additionally adjusted for diabetes and medications). Adherence to the Mediterranean diet was measured by the validated 14-item questionnaire.
